# Complementarity between the updated version of the front-of-pack nutrition label Nutri-Score and the food-processing NOVA classification

**DOI:** 10.1017/S1368980024000296

**Published:** 2024-02-01

**Authors:** Barthélemy Sarda, Emmanuelle Kesse-Guyot, Valérie Deschamps, Pauline Ducrot, Pilar Galan, Serge Hercberg, Melanie Deschasaux-Tanguy, Bernard Srour, Leopold K Fezeu, Mathilde Touvier, Chantal Julia

**Affiliations:** 1 Université Sorbonne Paris Nord and Université Paris Cité, INSERM, INRAE, CNAM, Nutritional Epidemiology Research Team (EREN), Center of Research in Epidemiology and StatisticS (CRESS), 74 rue Marcel Cachin, F-93017 Bobigny, France; 2 Nutritional Epidemiology Surveillance Team (ESEN), Santé publique France, The French Public Health Agency, Bobigny, France; 3 Santé publique France, French National Public Health Agency, Saint- Maurice, France; 4 Public health Department, Hôpital Avicenne, Assistance Publique-Hôpitaux de Paris (AP-HP), Bobigny, France

**Keywords:** Nutri-Score, Nutrient profiling system, NOVA, Food processing, Public health

## Abstract

**Objective:**

To compare the initial and the updated versions of the front-of-pack label Nutri-Score (related to the nutritional content) with the NOVA classification (related to the degree of food processing) at the food level.

**Design:**

Using the OpenFoodFacts database – 129,950 food products – we assessed the complementarity between the Nutri-Score (initial and updated) with the NOVA classification through a correspondence analysis. Contingency tables between the two classification systems were used.

**Settings:**

The food offer in France.

**Participants:**

Not applicable.

**Results:**

With both versions (i.e. initial and updated) of the Nutri-Score, the majority of ultra-processed products received medium to poor Nutri-Score ratings (between 77·9 % and 87·5 % of ultra-processed products depending on the version of the algorithm). Overall, the update of the Nutri-Score algorithm led to a reduction in the number of products rated A and B and an increase in the number of products rated D or E for all NOVA categories, with unprocessed foods being the least impacted (–3·8 percentage points (–5·2 %) rated A or B and +1·3 percentage points (+12·9 %) rated D or E) and ultra-processed foods the most impacted (–9·8 percentage points (–43·4 %) rated A or B and +7·8 percentage points (+14·1 %) rated D or E). Among ultra-processed foods rated favourably with the initial Nutri-Score, artificially sweetened beverages, sweetened plant-based drinks and bread products were the most penalised categories by the revision of Nutri-Score while low-sugar flavoured waters, fruit and legume preparations were the least affected.

**Conclusion:**

These results indicate that the update of the Nutri-Score reinforces its coherence with the NOVA classification, even though both systems measure two distinct health dimensions at the food level.

The Nutri-Score is a summary, graded, colour-coded front-of-pack nutrition label intended to inform consumers on the overall nutritional quality of foods, in an easy-to-understand format, to facilitate healthier choices during purchase. Based on a nutrient profiling system developed initially in the United Kingdom in 2005^(1)^, our research team as well as public health institutions designed jointly what became in 2017 the recommended front-of-pack nutrition labelling in France, that is, the Nutri-Score. Briefly, the evaluation provided by the Nutri-Score is based on the content per 100 g of product of unfavourable elements or nutrients (energy, simple sugars, saturated fatty acids and salt) and of favourable elements (proteins, fibre and proportion of fruit and vegetables) from the nutritional composition of foods. In the following years, other European countries endorsed the scheme and recommended its implementation on food packaging. Yet, discussions on the Nutri-Score continue as the European Commission is proposing to implement a harmonised and mandatory front-of-pack labelling scheme in the European Union^(2)^.

Among criticisms levelled against Nutri-Score, the lack of consideration for the degree of processing has been an argument to dismiss the label, as it does not base its assessment on this criterion. The degree of processing of foods is often defined using the NOVA classification, initially proposed by a research team from the University of Sao Paolo^(3)^. The notion of ‘ultra-processed foods’ originates from this system and relates to foods that have undergone intense industrial physical, chemical or biological processes (e.g. hydrogenation, moulding, extruding and pre-processing by frying) and/or that generally contain industrial substances not usually found in domestic kitchens (e.g. maltodextrin, hydrogenated oils or modified starches, flavouring agents, cosmetic additives: dyes, emulsifiers, non-nutritive sweeteners, etc.)^(4)^. Ultra-processed foods emerged in the 1950s in a context of globalisation of food systems, and since then have become a cornerstone of dietary patterns in industrialised countries, including Europe^(5)^. While their relative contribution in diets vary greatly among individuals and countries – reaching in some countries more than half of the energy contribution^(6)^ – their consumption has been on the rise globally, especially in low- and middle-income countries^(5)^. However, in the last decade, growing concerns have emerged over the consumption of such foods as research has shown that higher consumption of ultra-processed foods was associated with higher health risks, such as obesity^(7,8)^, type-2 diabetes^(9)^, cancer^(10)^ or all-cause mortality^(11,12)^. In light of this field of research, some countries started to recommend in their dietary guidelines to reduce the consumption of ultra-processed foods, including France.

On the other hand, front-of-pack nutrition labelling provides information to consumers as to the nutrient content of foods, as nutrient intakes have long been shown to impact long-term health^(13)^. Summary front-of-pack nutrition labellings use nutrient profiling to inform consumers on the overall nutritional quality of foods in a simplified indicator. Assessment provided by front-of-pack nutrition label, including the Nutri-Score, in most cases rely on macronutrient and/or micronutrient data (or proxies of them) and do not integrate the degree of processing in their evaluations.

While nutritional quality of foods and degree of processing covers two different health dimensions of foods, they are inter-related: on average, ultra-processed foods tend to be of lower nutritional quality with higher levels of energy, salt, sugar or fats^(14–16)^. Nevertheless, they are not collinear and correspond to complementary concepts at the level of a food product^(17)^. Some reconstituted meat substitutes or diet sodas may have a rather favourable nutritional profile per se (none-to-low calories and sugar, high content in legumes and vegetables), but they are typically ultra-processed (containing non-nutritive sweeteners, dyes, emulsifiers, etc.). Conversely, a home-made chocolate cake or a 100 % pure fruit juice are not ultra-processed but have a rather poor nutritional profile (high levels of saturated fats, sugar and/or energy). However, at the moment, only information on nutritional quality is directly available to consumers, through front-of-pack labelling, hence the importance of exploring the alignment between the two systems.

In 2023, an updated version of the nutrient profiling system used to compute the Nutri-Score was published^(18,19)^. The expert group in charge of the update proposed modifications to the calculation method with the aim of improving complementarity where necessary between Nutri-Score and food-based dietary guidelines^(20)^. The recent update of the system thus raises questions on the evolution of the classification of foods depending on their degree of processing considering that it has been a source of criticisms for the former version of the Nutri-Score. The aim of this study was to compare the classification of foods on French food market according to either NOVA classification and with both versions of the Nutri-Score (i.e. the initial and the updated versions).

## Methods

### Food composition data

Food composition from the French food market was based on the crowd-sourced food information database OpenFoodFacts^(21)^. The OpenFoodFacts project is an open sourced and collaborative database, listing products sold worldwide. The database provides data regarding the nutritional content of foods (mostly prepacked), based on the nutritional declaration displayed on the back-of-pack, the ingredient list and other information found on labels. Additionally, based on the list of ingredients, OpenFoodFacts provides the NOVA classification of the product. The analyses were conducted with data extracted in November 2021 and with products sold in France. The database cover extensively the French food market and includes 400 005 products.

Food categories not subject to display the Nutri-Score (e.g. alcoholic beverages, spices, dry tea, coffee or baby food) or foods with missing categorisation in the OpenFoodFacts database were excluded. Missing values were treated as follows:

- In case of missing values or outlier values for a mandatory nutrient according to the EU *n* 1169/2011 Regulation or NOVA classification, products were excluded;
- In case of missing fibre values, for products belonging to a group with no fibre (e.g. fish and seafood, fats and oils), a value of 0 g was imputed. For products belonging to groups with a significant amount of fibre (defined as a group for which at least 25 % of products with fibre information contained more than 0.9 g of fibre (the first threshold at which the Nutri-Score allocates points for fibre)), products were excluded;
- In case of missing values for the fruit and vegetable component, three possibilities were considered. First, if a product belonged to a group with no apparent element from fruit or vegetable component, 0 % was imputed to all the products with missing values in that category (e.g. pasta, bread, rice, fish or meat). Then, for mono-ingredient products in categories belonging to the fruit and vegetable component (e.g. fruit, vegetables or fruit juices), 100 % was imputed. Other products were excluded.

**Fig. 1:**
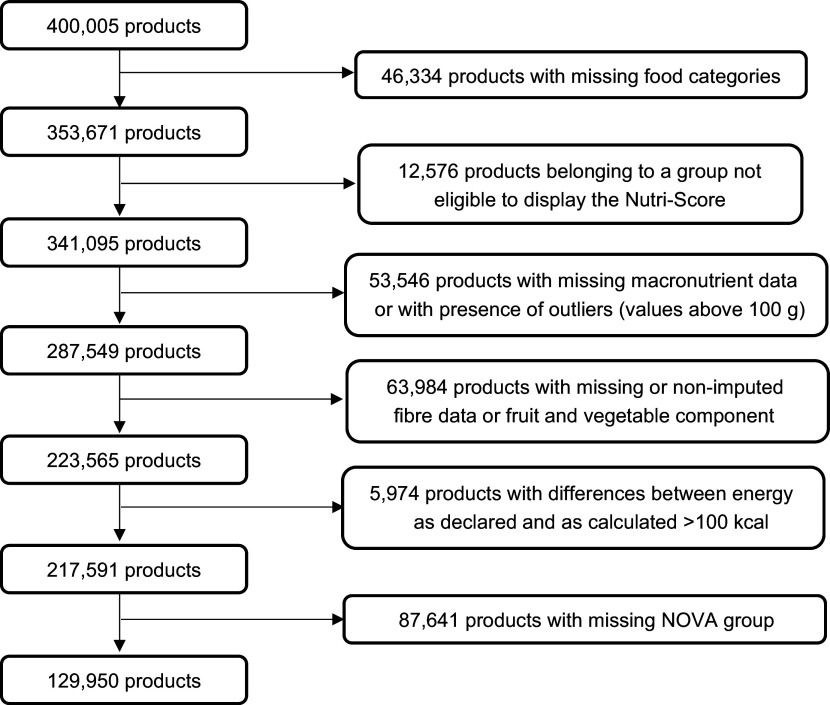
Flow chart of the products included in the study

A flow chart corresponding to the data management is presented below (Fig. 1).

A list of the food groups and sample size of the final sample is presented in see online supplementary material, Supplementary Material 1.

Finally, NOVA classification was checked using the food categories, product names, ingredients lists as well as the list of additives to correct potential misclassification, according to published guidelines^(4)^. The list of additives was used to detect if products were inadequately classified as NOVA 1 as in this category products may only contain some specific preservatives. The food categories (and if necessary product names and ingredient lists) were used to detect and to reclassify if there were any outliers (e.g. prepared dishes in the NOVA 1 category or 100 % fruit juices in the NOVA 4 categories).

### Nutri-Score computation

The Nutri-Score was computed using the guidelines provided by the French Public Health Agency for the initial and updated version of the algorithm^(22)^.

To explain briefly, the initial score computation system bases its assessment on the nutritional composition of the food as sold per 100 g (or 100 ml for beverages): on one hand, points are allocated for the ‘negative’ components (energy (kJ), saturated fat (g), sugar (g) and salt (g) or Na (mg)) and on the other hand points are allocated for the ‘favourable’ components (protein (g), fibre (g), fruits/vegetables/pulses/nuts and oils (%)). Using the two subtotals, a final score is computed, and a rating is then assigned based on the final score.

The updated version of the algorithm functions similarly to its predecessor. There have been adaptations such as increase of the maximum number of points for the sugar and salt components, which led to less favourable ratings for foods with high content in sugar or in salt. Then, points pertaining to the fibre component and the protein component were more strictly allocated, meaning that a greater content of fibre or protein was needed to receive points from the ‘favourable’ component. Additionally, specific rules were added for red meat, which limited the maximum number of points red meat products could from the ‘favourable’ component; in beverages, the presence of non-nutritive sweetener was added as an additional ‘unfavourable’ element. Finally, threshold to convert the final score into the rating was adapted to the new algorithm. Detailed description of the initial and updated version of the Nutri-Score can be found in see online supplementary material, Supplemental Material 2.

### NOVA classification

The degree of processing was assessed using the NOVA classification, which consists of four groups. First, NOVA 1 group includes unprocessed or minimally processed foods (fresh, dried, grounded, chilled, frozen, pasteurised or fermented staple foods such as fruits, vegetables, pulses, rice, pasta, eggs, meat, fish or milk). Then, NOVA 2 group includes processed culinary ingredients (salt, vegetable oils, butter, sugar and other substances extracted from foods and used in kitchens to transform unprocessed or minimally processed foods into culinary preparations) Then, NOVA 3 group includes processed foods (canned vegetables with added salt, sugar-coated dry fruits, meat products only preserved by salting, cheeses and freshly made unpackaged breads and other products manufactured with the addition of salt, sugar or other substances of the ‘processed culinary ingredients’ group). Finally, NOVA 4 group includes ultra-processed foods (i.e. products that undergo industrial processes that include for instance hydrogenation, hydrolysis, extruding, moulding, reshaping and pre-processing by frying). Flavouring agents, colours, emulsifiers, humectants, non-nutritive sweeteners and other cosmetic additives are often added to NOVA 4 products to imitate sensorial properties of unprocessed or minimally processed foods and their culinary preparations. The ultra-processed foods group is defined by opposition to the three other NOVA groups.

The assessment of the NOVA classification was conducted by the OpenFoodFacts database based on the list of ingredients and food categorisation, as explained the OpenFoodFacts website^(23)^. To explain briefly, all products are by default classified NOVA 1 and if products contain qualifying ingredients, their classification is changed. For example, food items containing ingredients exclusively found in NOVA 4 products (e.g. cosmetic additives (food dyes, emulsifiers, texture agents, etc.), flavouring agents and hydrogenated oils) are automatically classified as ultra-processed. Products in the NOVA group 2–4 tend to be accurately classified as qualifying ingredients were used to determine their classification. As a result, we considered that the main source of error would be in case of undetected ingredients and thus the NOVA 1 group was the most likely to contain misclassified products. We controlled the quality of products classified as NOVA 1 based on the food categorisation and the list of ingredients and manually reclassified errors in the adequate NOVA group (723 products (5 % of NOVA 1 products) were reclassified following this procedure).

### Statistical analysis

Results are presented as frequencies of products in each Nutri-Score group per NOVA group. Differences in the distribution across categories for each NOVA group were tested using *χ* ^2^ tests.

While the Nutri-Score does not have the purpose to classify foods as healthy or unhealthy, but rather to indicate which products are of higher or lower nutritional quality in comparison with other products of the same category we investigated which food groups corresponded to less coherent ratings (i.e. favourable (A and B) ratings for NOVA 4 products and unfavourable ratings (D and E) for NOVA 1) products.

All statistical analyses were performed with SAS software, version 9.4 (SAS Institute Inc., Cary, NC, USA).

## Results

The final database included 129 950 products from the French food market, for which both versions of the Nutri-Score were computed. Among these products, NOVA 4 products were the most represented category (*n* 79 512; 61 % of all products), followed by NOVA 3 (*n* 33 255; 26 % of all products) and NOVA 1 products (*n* 14 073; 11 % of all products), and finally NOVA 2 products (*n* 3110; 2 % of all products). Of note, the focus of the Open Food Facts database on prepacked foods explains the predominance of processed and ultra-processed foods.

Table 1 displays the distribution of the classification of different NOVA groups according to both versions of the Nutri-Score. With both versions of the Nutri-Score, NOVA 1 products were rated in majority A or B (initial algorithm: 72·5 % and updated algorithm: 68·7 %), and a limited proportion was rated D or E (initial algorithm: 9·9 % and updated algorithm: 11·4 %). The most represented Nutri-Score categories for NOVA 4 (resp. NOVA 3) products were the D or E categories (initial algorithm: 55·4 % and updated algorithm: 63·2 % (resp. 48·1 % and 53·9 %)), and a smaller proportion was rated A or B (initial algorithm: 22·1 % and updated algorithm: 12·5 % (resp. 32·9 % and 25·6 %)). Overall, the update of the Nutri-Score algorithm led to a lower proportion of products in A and B and a higher proportion of products in D or E categories for all NOVA categories, with notable differences between groups (NOVA 1: –3·8 percentage points products rated A or B (–5·2 %) and +1·3 percentage points (+12·9 %) products rated D or E; NOVA 3: –7·3 percentage points (–22·2 %) products rated A or B and +5·8 percentage points (+12·1 %) products rated D or E ; NOVA 4:–9·6 percentage points (–43·4 %) products rated A or B and +7·8 percentage points (+14·1 %) products rated D or E). The relative increase in strictness (i.e. less product rated A or B and more rated D or E) was also observed across all the database: regardless of the food group, 29 % of products in our database saw their rating being deteriorated, 6 % of products in our database saw their rating being improved and 65 % of products had the same rating.

**Table 1:** Cross-frequency table between Nutri-Score and NOVA classification

Group	***n***		A	B	C	D	E	*P*-value*
**NOVA 1** †	14 073	**Initial**	56·3 %	16·2 %	17·4 %	4·9 %	5·2 %	<0·001
		**Updated**	50·1 %	18·6 %	19·9 %	7·5 %	3·9 %	
**NOVA 2**	3110	**Initial**	0·7 %	3·1 %	26·6 %	38·4 %	31·2 %	<0·001
		**Updated**	2·7 %	18·5 %	15·7 %	10·5 %	52·6 %	
**NOVA 3**	33 255	**Initial**	19·0 %	13·9 %	19·0 %	33·8 %	14·3 %	<0·001
		**Updated**	16·2 %	9·4 %	20·5 %	30·6 %	23·3 %	
**NOVA 4**	79 512	**Initial**	8·6 %	13·5 %	22·5 %	31·8 %	23·6 %	<0·001
		**Updated**	5·3 %	7·2 %	24·3 %	29·3 %	33·9 %	

* *P* values correspond to *χ* ^2^ tests between the initial and updated classification using the Nutri-Score. A test has been performed for each NOVA category.

† Relative frequencies were calculated by rows.

In Table 2, NOVA 1 products with a D or E and NOVA 4 products with A or B, considered as less coherent, are presented for each version of the algorithm. Regardless of the version of the algorithm used, most of NOVA 1 products rated D or E were fruit juices (86 % with the initial algorithm and 79 % with the updated algorithm). With the updated algorithm, the proportion of fruit juices rated D or E slightly increased (from 33·3 % to 34·1 %), while the number of dried fruits unfavourably rated was doubled – due to their higher content in sugar – leading to 22·8 % of dried fruits being rated D or E. For NOVA 4 products, ready-to-eat meals were the most represented category in A or B with the initial algorithm, followed by dairy products (i.e. yogurts or milk-based beverages), bread products (i.e. sandwich bread or rusks) and vegetable preparations (i.e. canned vegetables or salads with added additives). The impact of the update of the Nutri-Score algorithm varied greatly across categories. The categories with the greatest decline in the proportion of NOVA 4 products in A or B categories between the initial and updated algorithm were the following: bread products (–70·3 %), ready-to-eat dishes (–50·1 %), milk and yogurts (–55·0 %), plant-based beverages (–75·6 %), breakfast cereals (–50·3 %) and artificially sweetened beverages (–93·9 %). On the contrary, flavoured waters was the only category for which the number of NOVA 4 products rated A or B increased (+86·5 %) and fruit products and prepared legumes (e.g. canned or plant-based meat alternatives with legumes) were the least negatively affected categories (resp. –7·1 % and –7·0 %).

**Table 2:** Description and evolution of NOVA 1 products rated D or E and NOVA 4 products rated A or B

NOVA 1		NOVA 1 products rated D or E					
Group	*n*	Indicative foods	Initial Nutri-Score		Updated Nutri-Score		Relative variation
			*n*	%	*n*	%	
Fruit juices*	3668	Fruit juices	1220	33·3 %	1252	34·1 %	+2·6 %
Dried fruits	556	Dried coconut, goji berries…	60	10·8 %	127	22·8 %	+112 %
Meat	607	Cuts of red meat high in saturated fat	57	10·3 %	61	10·9 %	+7·0 %
Plant-based beverages	118	Coconut milk	49	41·5 %	60	50·8 %	+18·3 %

NOVA 4		NOVA 4 products rated A or B					
Group	*n*	Indicative foods	Initial Nutri-Score		Updated Nutri-Score		Relative variation
			*n*	%	*n*	%	
Ready-to-eat dishes	6828	All types of ready-to-eat dishes	4533	66·4 %	2260	33·1 %	−50·1 %
Vegetable-based or vegetarian dishes	3174	Vegetable salads, sandwiches, soups…	1983	63·5 %	1017	32·0 %	−48·7 %
Meat-based dishes	1747	Cassoulet, sausage and lentils, shepherd’s pie…	1020	58·3 %	350	20·0 %	−65·7 %
Poultry-based dishes	921	Curry, paella with chicken…	827	89·8 %	476	51·7 %	−42·4 %
Fish-based dishes	986	Fish with rice, breaded fish…	703	71·3 %	375	38·0 %	−46·7 %
Milk and yogurts	3990	Flavoured yogurts, flavoured milks	1813	45·4 %	815	20·4 %	−55·0 %
Bread	2232	Sandwich bread, rusks…	1461	65·5 %	434	19·4 %	−70·3 %
Vegetables	942	Prepared vegetables (canned or fresh)	845	89·7 %	746	79·2 %	−11·7 %
Meat	2666	Poultry preparations	722	27·1 %	641	24·0 %	−11·2 %
Fish	1567	Canned fish, breaded fish	669	42·7 %	554	35·4 %	−17·2 %
Cereal products	1257	Prepared pasta (plain or stuffed)	650	51·7 %	353	28·1 %	−45·7 %
Artificially sweetened beverages	1889	Soft drinks sweetened with non-nutritive sweeteners	478	25·3 %	29	1·5 %	−93·9 %
Flavoured waters	1111	Flavoured waters unsweetened or lightly sweetened	342	30·8 %	638	57·4 %	86·5 %
Plant-based beverages	727	Plant-based milks	569	78·3 %	139	19·1 %	−75·6 %
Sauces, dressings and dips	4596	Tomato-based sauces, guacamole…	565	12·2 %	374	8·1 %	−33·8 %
Sweets	10 430	Plant-based yogurts, jam low in sugar	531	5·1 %	360	3·5 %	−32·2 %
Soups	728	Soups	526	72·3 %	348	47·8 %	−33·8 %
Breakfast cereals	1701	Muesli, whole-grain granola	441	25·9 %	219	12·9 %	−50·3 %
Fruits	520	Compotes, fruit preparations	422	81·1 %	392	75·4 %	−7·1 %
Processed meat	6918	Ham	354	5·1 %	229	3·3 %	−35·3 %
Legumes	345	Canned and/or prepared legumes	284	82·3 %	264	76·5 %	−7·0 %

Categories representing less than 2 % of the total number of NOVA 1 products rated D or E (resp. NOVA 4 products rated A or B) were not presented.

* The results should be read as followed: ‘Out of the 3668 products categorised as fruit juices and as NOVA 1 products, 1220 products were rated D or E with the initial Nutri-Score and 1252 with the updated algorithm, representing a relative increase of 2.6 %’.

## Discussion

The present study shows for the first time the effect of the update of the Nutri-Score’s algorithm on the alignment between the Nutri-Score and the NOVA classification. The results show that the initial Nutri-Score was overall aligned with the NOVA classification by rating favourably most unprocessed products and unfavourably the majority of ultra-processed products, even though some discrepancies were observable. The update of the Nutri-Score allowed to further align these two classifications given that ultra-processed products were the most penalised NOVA group by the update and unprocessed products were little affected.

To the authors’ knowledge, few studies explored the alignment between the NOVA classification and the initial version of the Nutri-Score on large branded food composition databases^(24,25)^, even though other studies have investigated specifics sectors^(26–29)^ or a more limited selection of foods^(30)^. All studies that investigated the food offer cross-sectionally, including the present one, found that between 51·5 % and 56·0 % of NOVA 4 products were rated D or E, with the initial version of the Nutri-Score, showing that results were consistent across studies. Overall, a minority of ultra-processed products are rated A or B, which is unsurprising as ultra-processed products tend to have higher content in fat, simple sugars and/or salt^(14–16)^, and thus are poorly rated by the Nutri-Score. Interestingly, the study from Romero Ferreiro et al.^(25)^ used the relative frequency of NOVA groups per Nutri-Score category as its main outcome. Given the clear imbalance in proportions on the food market between NOVA groups – 56 % of products in the study from Romero Ferreiro were NOVA 4 – the results obtained by Romero Ferreiro et al. reflect the preponderance of ultra-processed products on the market rather than the actual ability of the Nutri-Score to discriminate products from different NOVA categories. Thus, in this study, the choice was made to use the relative frequency of Nutri-Score categories per NOVA groups.

Nonetheless, a significant proportion of NOVA 4 products was rated in the A and B categories. Indeed the Nutri-Score and the NOVA classification cover two distinct but complementary aspects of the health dimensions of foods^(24,25)^. The Nutri-Score objective is to describe and summarise in a clear and understandable format the nutritional quality of foods. Some foods classified as NOVA 4 may have a favourable nutritional profile (e.g. unsweetened artificially flavoured water, some canned legumes with added additives or plant-based meat substitutes using emulsifiers and/or texturing agents). The Nutri-Score rated unfavourably the majority of ultra-processed foods in relation to their poor nutritional profile, without considering their degree of processing. Indeed, the Nutri-Score does not include other aspects that may also be relevant to evaluate the overall health effects of an individual food, such as degree of processing or formulation (i.e. presence of food additives), contamination by packaging, or pesticide content. However, given the current state of evidence, it has not been possible to elaborate a comprehensive indicator that would integrate all different health dimensions. As such, the Nutri-Score ‘only’ provides information on nutritional quality, the importance of which for long-term health has been demonstrated by decades of research and is acknowledged by national and international health authorities^(31,32)^. Numerous studies have also demonstrated that diets composed of products with a more favourable rating by the initial version of the Nutri-Score was associated with a lesser risk of developing chronic diseases^(33–36)^ and mortality^(37,38)^ in various cohorts and contexts.

Overall, the update of the Nutri-Score algorithm led to a reduction in the number of products rated A and B and an increase in the number of products rated D or E for all NOVA categories, with unprocessed foods being the least impacted and ultra-processed foods the most impacted. Regarding unprocessed and minimally processed products, the rather constant number of products rated D and E is mostly attributable to the stable number of fruit juices rated D or E, which represent the vast majority of NOVA 1 products rated D or E in our data (86 % with the initial algorithm and 80 % with updated algorithm). For ultra-processed products, the number of products rated A or B decreased for all categories except for flavoured waters. Most heavily impacted categories were those which benefited with the initial Nutri-Score from points compensation between the favourable and unfavourable component (i.e. bread products, ready-to-eat dishes, flavoured yogurts and breakfast cereals), mainly due to the relatively lenient thresholds for the fibre and protein component. Ready-to-eat dishes are typical of this phenomenon, as they are mix of different foods (usually a source of protein, vegetables and a cereal), they tend contain intermediary content of many nutrients/ or elements, which tend to be rated favourably with more lenient thresholds. The use of stricter thresholds in the favourable component impacted considerably their ratings. On the other hand, prepared vegetables, fruits or legumes – which were classified as ultra-processed because of added additives such as artificial flavours, texturing agents or emulsifiers – were the least affected categories as their favourable rating stemmed from their low content in ‘unfavourable’ components (energy, saturated fat and sugar or salt). For beverages, several modifications explain the results observed. First, a specific ‘unfavourable’ component has been implemented for the addition of non-nutritive sweeteners, which led to the almost absence of artificially sweetened beverages in the B category. Then, change in calculation’s modalities for dairy and plant-based beverages deteriorated the rating of any product with added sugar (e.g. chocolate milk or sweetened almond drink). Finally, the classification became slightly more lenient for very-low-sugar beverages (less than 2 g of sugar per 100 ml), hence the increase in the number of flavoured waters rated B.

In the last decade, the body of evidence regarding the degree of food processing increased dramatically, with links established between high consumption of ultra-processed foods and chronic diseases^(9,10)^ and all-cause mortality^(11,39–41)^. However, the relative importance of nutrient intake *v*. ultra-processed food consumption on health is still underexplored in the scientific literature. In a recent study from the NutriNet-Santé cohort, we showed that the overall quality of the diet at the individual level was attributable to ultra-processing and nutritional quality of foods in similar proportions (resp. 30 % and 26 %), but 44 % of the effect was attributed to cross-effects between the two^(42)^. These first results support the hypothesis that nutritional quality and ultra-processing are indeed two complementary but distinct dimensions of diet quality, but such results need to be replicated in other contexts and mechanisms involved should be investigated.

At this point, it should be pointed out that most front-of-pack nutrition labelling systems implemented in the world inform on nutritional quality and none inform on the degree of processing. Systems, such as Multiple Traffic Lights, the Health Star rating or Nutr’Inform Battery, do not integrate degree of processing in their assessment. Additionally, though warning labels – implemented in various countries in South America – are only displayed on processed and ultra-processed foods, a study found that 33·2 % of NOVA 4 products of the Chilean basic food basket would not display any warning^(30)^. Thus, most front-of-pack labels distinguish products from varying nutritional quality, even among ultra-processed foods. This relates to the availability from a regulatory perspective of data on the nutritional content of foods, through international guidelines from Codex Alimentarius, which support the implementation of front-of-pack nutrition labellings based on this information. For ultra-processed foods, no such regulatory definition yet exists, which may limit the ability of governments to draft legislation due to risks of challenges through the courts by manufacturers. While such a definition would be the basis to support regulation, pushing this issue on the agenda of the global governance on nutritional labelling (Codex Alimentarius) would require a modification of the power balance in this institution between public and private actors^(43,44)^. Finally, it should also be noted some label initiatives that aim to inform first and foremost on food processing (e.g. Siga classification) have been developed as online information, but have been little investigated and validated.

Nevertheless, in a recent randomised control trial, we investigated the impact of adding a black banner on the Nutri-Score for ultra-processed foods on the objective understanding of French consumers and found that this combined graphical format allowed consumers to both identify products of better nutritional quality and ultra-processed foods^(45)^. However, little is still known on the relative health impact of each dimension. Thus, additional scientific knowledge is needed to better inform consumers on how to prioritise products in case of conflicting characteristics (e.g. (un)processed foods of poor nutritional quality *v*. ultra-processed food of better nutritional quality) and how this arbitration may impact health in the long term. Another solution may be to integrate the degree of processing in the underlying nutrient profiling system, which would allow to keep the label easy to understand and would avoid information overload^(46)^. However, progress in our understanding of the interaction between nutritional quality and the degree of processing is required before being able to combine both dimensions in a single indicator.

The present work is the first one to report data on the recent update of the Nutri-Score and its impact on the correlation between the Nutri-Score and the NOVA classification. The use of the OpenFoodFacts database allowed us to have access to a wide range of prepacked products with 129 950 products analysed, with their corresponding NOVA classification. However, it should be pointed out that the main strength of the study is also its main limitation: the use of the Open Food Facts database. Although the Open Food Facts database includes a large number of products available on the market, we have no information about the representativeness of the sample of foods retrieved, either in terms of number of products or market share. However, to our knowledge, such a comprehensive database, detailing food composition, NOVA and food group classification and sales volumes does not exist with a similar degree of coverage to that provided by the OpenFoodFacts database even after considering the number of products with missing data (32·5 % of products had complete information for our study). Furthermore, as with any contributor-based database, some errors in food composition or classification could not be excluded. However, even if contributors are subject to errors, the errors are minimised by text and image recognition algorithms enabling automated checks. In addition, systematic control campaigns including random sampling and control of food products as well as updating of information are regularly carried out. Then, for the qualification of the degree of processing, we used the NOVA classification, which has been largely used to conduct research but has received criticisms for its lack of robustness and the lack of consideration for food science knowledge^(47,48)^. The attribution of NOVA group for each product was realised in the OpenFoodFacts database based on a textual analysis of the list of ingredients and the food categorisation, which may have led to inaccuracies even though manual checks were conducted to minimise errors. Then, the OpenFoodFacts database mainly contains pre-packed products sold in supermarkets, which means that products sold in bulk are under-represented. This phenomenon disproportionately affects NOVA 1 products – mainly fruit, vegetables, raw meat and fish – which may explain the high proportion of NOVA 3 and 4 products in the database. However, as we have seen in the present study, unprocessed products with no addition of fat, sugar and salt are rated favourably by the Nutri-Score, and thus we can hypothesise that the results would be similar if they were included. Finally, the approach with ultra-processed foods is binary (i.e. consumption of ultra-processed foods should be limited *v*. foods from other NOVA categories which should privileged over ultra-processed alternatives), whereas the Nutri-Score proposes a gradual scale, which required us to decide subjectively which categories of the Nutri-Score were considered aligned with the different NOVA categories. As there is no gold standard method for such analyses, we based our decision on the Nutri-Score’s colour code and implicit meaning (i.e. A and B are dark and light green, signalling foods that should be privileged; D and E are light and dark orange, signalling foods that should be limited). While this decision could be discussed, to our knowledge, no data indicate which Nutri-Score category would be perceived as discordant by consumers for each respective NOVA categories.

To conclude, the updated version of the Nutri-Score appeared to be more aligned with the NOVA classification, with significantly less ultra-processed foods being rated favourably. The Nutri-Score and the NOVA classification cover complementary but distinct dimensions at the food level. Further research needs to identify the mechanisms by which ultra-processed foods affect health and how they interact with nutritional quality. Such information is required if we want in the future to further upgrade the algorithm of the Nutri-Score by integrating both dimensions. Meanwhile, health authorities should communicate on how to use the Nutri-Score adequately, while promoting the consumption of minimally processed foods.

## Supporting information

Sarda et al. supplementary material Sarda et al. supplementary material

## Data Availability

The OpenFoodFacts data used in the study is available on their website (https://world.openfoodfacts.org/, accessed on November 2021) OpenFoodFacts is an open collaborative database of food products marketed worldwide, licensed under the Open Database License (ODBL).

## Supplementary material

For supplementary material accompanying this paper visit https://doi.org/10.1017/S1368980024000296
