# Supplementary material for: Complementarity between the updated version of the front-of-pack nutrition label Nutri-Score and the food-processing NOVA classification
Source: Public Health Nutr. 2024 Feb 1;27(1):e63. doi: 10.1017/S1368980024000296 (PMC10897572; doi:10.1017/S1368980024000296)
Supplement: Sarda et al. supplementary material [file S1368980024000296sup001.docx]

Supplementary Material 1: Food groups and sample size of products included in the study

| Food groups | Sample size | Proportion |
| --- | --- | --- |
| Appetizers | 2,884 | 2.22% |
| Artificially sweetened beverages | 1,892 | 1.46% |
| Biscuits and cakes | 9,292 | 7.15% |
| Bread | 3,143 | 2.42% |
| Breakfast cereals | 2,702 | 2.08% |
| Cereal r | 5,427 | 4.18% |
| Cheese | 7,332 | 5.64% |
| Chocolate products | 2,502 | 1.93% |
| Dairy desserts | 3,013 | 2.32% |
| Dressings and sauces | 7,272 | 5.60% |
| Dried fruits | 1,344 | 1.03% |
| Eggs | 466 | 0.36% |
| Fats | 2,368 | 1.82% |
| Fish and seafood | 5,063 | 3.90% |
| Fruit juices | 8,925 | 6.86% |
| Fruit nectars | 121 | 0.09% |
| Fruits | 2,196 | 1.69% |
| Ice cream | 2,495 | 1.92% |
| Legumes | 1,447 | 1.11% |
| Meat | 3,670 | 2.82% |
| Milk and yogurt | 6,210 | 4.78% |
| Nuts | 1,415 | 1.09% |
| Offals | 430 | 0.33% |
| One-dish meals | 8,980 | 6.91% |
| Pastries | 1,734 | 1.33% |
| Pizza pies and quiches | 1,320 | 1.02% |
| Plant-based milk substitutes | 1,125 | 0.87% |
| Potato product | 769 | 0.59% |
| Processed meat | 8,348 | 6.42% |
| Salty and fatty products | 4,793 | 3.69% |
| Sandwiches | 795 | 0.61% |
| Soups | 1,233 | 0.95% |
| Sweets | 12,311 | 9.47% |
| Unsweetened beverages | 2,529 | 1.95% |
| Vegetables | 3,892 | 2.99% |
| Waters and flavored waters | 512 | 0.39% |

Supplementary Material 2: Computation of the Nutri-Score

1. **The 2017 Nutri-Score**

Points are allocated based on the nutritional content per 100g of foods or beverages.

Points are allocated for unfavourable elements (A points) and for favourable elements (C points)

| Component | A points | | | | C points | | |
| --- | --- | --- | --- | --- | --- | --- | --- |
| Points | Energy (kJ) | Saturated fat (g) | Sugars (g) | Sodium (mg) | Protein (g) | Fibre (g) | Fruits, vegetables, pulses (%) |
| 0 | ≤335 | ≤1 | ≤4.5 | ≤90 | ≤1.6 | ≤0.9 | ≤40% |
| 1 | >335 | >1 | >4.5 | >90 | >1.6 | >0.9 | >40% |
| 2 | >670 | >2 | >9 | >180 | >3.2 | >1.9 | >60% |
| 3 | >1005 | >3 | >13.5 | >270 | >4.8 | >2.8 |  |
| 4 | >1340 | >4 | >18 | >360 | >6.4 | >3.7 |  |
| 5 | >1675 | >5 | >22.5 | >450 | >8.0 | >4.7 | >80% |
| 6 | >2010 | >6 | >27 | >540 |  |  |  |
| 7 | >2345 | >7 | >31 | >630 |  |  |  |
| 8 | >2680 | >8 | >36 | >720 |  |  |  |
| 9 | >3015 | >9 | >40 | >810 |  |  |  |
| 10 | >3350 | >10 | >45 | >900 |  |  |  |

A points= Points_energy_+ Points_Saturated fat_+ Points_Sugars_+ Points_Salt_

C points= Points_Fiber_+ Points_Protein_+ Points_F,V,P_

**In the general case:**

- If A points<11; Final nutritional score (FNS)=A points – C points
- If A≥11, FNS= A points – (Points_Fiber_ + Points_F,V,P_)

**Specific calculation rules:**

1. For cheeses:

Same calculations as in the general case except for the FNS

- FNS=A points – C points (regardless of the value of A)

1. For fats and oils:

| Component | A points | | | | C points | | |
| --- | --- | --- | --- | --- | --- | --- | --- |
| Points | Energy (kJ) | Ratio SFA/total fat (%) | Sugars (g) | Sodium (mg) | Protein (g) | Fibre (g) | Fruits, vegetables, pulses (%)^1^ |
| 0 | ≤335 | <10 | ≤4.5 | ≤90 | ≤1.6 | ≤0.9 | ≤40% |
| 1 | >335 | >10 | >4.5 | >90 | >1.6 | >0.9 | >40% |
| 2 | >670 | >16 | >9 | >180 | >3.2 | >1.9 | >60% |
| 3 | >1005 | >22 | >13.5 | >270 | >4.8 | >2.8 |  |
| 4 | >1340 | >28 | >18 | >360 | >6.4 | >3.7 |  |
| 5 | >1675 | >34 | >22.5 | >450 | >8.0 | >4.7 | >80% |
| 6 | >2010 | >40 | >27 | >540 |  |  |  |
| 7 | >2345 | >46 | >31 | >630 |  |  |  |
| 8 | >2680 | >52 | >36 | >720 |  |  |  |
| 9 | >3015 | >58 | >40 | >810 |  |  |  |
| 10 | >3350 | ≥64 | >45 | >900 |  |  |  |

^1^ Olive, canola and walnut oil are included in the fruit and vegetable component

A points= Points_energy_+ Points_Saturated fat_+ Points_Sugars_+ Points_Salt_

C points= Points_Fiber_+ Points_Protein_+ Points_F,V,P_

- If A points<11; Final nutritional score (FNS)=A points – C points
- If A≥11, FNS= A points – (Points_Fiber_ + Points_F,V,P_)

1. Beverages

| Component | A points | | | | C points | | | |
| --- | --- | --- | --- | --- | --- | --- | --- | --- |
| Points | Energy (kJ) | Saturated fat (g) | Sugars (g) | Sodium (mg) | | Protein (g) | Fibre (g) | Fruits, vegetables, pulses (%) |
| 0 | ≤0 | ≤1 | ≤0 | ≤90 | | ≤1.6 | ≤0.9 | ≤40% |
| 1 | >0 | >1 | >0 | >90 | | >1.6 | >0.9 | >40% |
| 2 | >30 | >2 | >1.5 | >180 | | >3.2 | >1.9 | >60% |
| 3 | >60 | >3 | >3 | >270 | | >4.8 | >2.8 |  |
| 4 | >90 | >4 | >4.5 | >360 | | >6.4 | >3.7 |  |
| 5 | >120 | >5 | >6 | >450 | | >8.0 | >4.7 | >80% |
| 6 | >150 | >6 | >7.5 | >540 | |  |  |  |
| 7 | >180 | >7 | >9 | >630 | |  |  |  |
| 8 | >210 | >8 | >10.5 | >720 | |  |  |  |
| 9 | >240 | >9 | >12 | >810 | |  |  |  |
| 10 | >270 | >10 | >13.5 | >900 | |  |  |  |

A points= Points_energy_+ Points_Saturated fat_+ Points_Sugars_+ Points_Salt_

C points= Points_Fiber_+ Points_Protein_+ Points_F,V,P_

- If A points<11; Final nutritional score (FNS)=A points – C points
- If A≥11, FNS= A points – (Points_Fiber_ + Points_F,V,P_)

Nutri-Score allocation

| FNS | | Class |
| --- | --- | --- |
| All foods except beverages | Beverages |  |
| ≤-1 | Waters | A |
| 0 to 2 | ≤1 | B |
| 3 to 10 | 2 to 5 | C |
| 11 to 18 | 6 to 9 | D |
| ≥19 | ≥19 | E |

1. **The 2023 Nutri-Score**

Points are allocated based on the nutritional content per 100g of foods or beverages.

Points are allocated for unfavorable elements (A points) and for favorable elements (C points)

| Component | A points | | | | C points | | |
| --- | --- | --- | --- | --- | --- | --- | --- |
| Points | Energy (kJ) | Saturated fat (g) | Sugars (g) | Salt (g) | Protein (g) | Fiber (g) | Fruits, vegetables, pulses (%) |
| 0 | ≤335 | ≤1 | ≤3.4 | ≤0.2 | ≤2.4 | ≤3 | ≤40% |
| 1 | >335 | >1 | >3.4 | >0.2 | >2.4 | >3 | >40% |
| 2 | >670 | >2 | >6.8 | >0.4 | >4.8 | >4.1 | >60% |
| 3 | >1005 | >3 | >10 | >0.6 | >7.2 | >5.2 |  |
| 4 | >1340 | >4 | >14 | >0.8 | >9.6 | >6.3 |  |
| 5 | >1675 | >5 | >17 | >1 | >12 | >7.4 | >80% |
| 6 | >2010 | >6 | >20 | >1.2 | >14 |  |  |
| 7 | >2345 | >7 | >24 | >1.4 | >17 |  |  |
| 8 | >2680 | >8 | >27 | >1.6 |  |  |  |
| 9 | >3015 | >9 | >31 | >1.8 |  |  |  |
| 10 | >3350 | >10 | >34 | >2.0 |  |  |  |
| 11 |  |  | >37 | >2.2 |  |  |  |
| 12 |  |  | >41 | >2.4 |  |  |  |
| 13 |  |  | >44 | >2.6 |  |  |  |
| 14 |  |  | >48 | >2.8 |  |  |  |
| 15 |  |  | >51 | >3 |  |  |  |
| 16 |  |  |  | >3.2 |  |  |  |
| 17 |  |  |  | >3.4 |  |  |  |
| 18 |  |  |  | >3.6 |  |  |  |
| 19 |  |  |  | >3.8 |  |  |  |
| 20 |  |  |  | >4 |  |  |  |

A points= Points_energy_+ Points_Saturated fat_+ Points_Sugars_+ Points_Salt_

C points= Points_Fiber_+ Points_Protein_+ Points_F,V,P_

**In the general case:**

- If A points<11; Final nutritional score (FNS)=A points – C points
- If A≥11, FNS= A points – (Points_Fiber_ + Points_F,V,P_)

**Specific calculation rules:**

1. For cheeses:

Same calculations as in the general case except for the FNS

- FNS==A points – C points (regardless of the value of A)

1. For red meat and processed meat:

Same calculations as in the general case except maximum number of protein points is set at 2.

- If A points<11; FNS=A points – (Points_Fiber_+ min(Points_Protein_,2)+ Points_F,V,P_)
- If A≥11, FNS= A points – (Points_Fiber_ + Points_F,V,P_)

1. For nuts, seeds and oils:

| Component | A points | | | | C points | | |
| --- | --- | --- | --- | --- | --- | --- | --- |
| Points | Energy from SFA (kJ)^1^ | Ratio SFA/total fat (%) | Sugars (g) | Salt (g) | Protein (g) | Fiber (g) | Fruits, vegetables, pulses (%)^2^ |
| 0 | ≤120 | <10 | ≤3.4 | ≤0.2 | ≤2.4 | ≤3 | ≤40% |
| 1 | >120 | >10 | >3.4 | >0.2 | >2.4 | >3 | >40% |
| 2 | >240 | >16 | >6.8 | >0.4 | >4.8 | >4.1 | >60% |
| 3 | >360 | >22 | >10 | >0.6 | >7.2 | >5.2 |  |
| 4 | >480 | >28 | >14 | >0.8 | >9.6 | >6.3 |  |
| 5 | >600 | >34 | >17 | >1 | >12 | >7.4 | >80% |
| 6 | >720 | >40 | >20 | >1.2 | >14 |  |  |
| 7 | >840 | >46 | >24 | >1.4 | >17 |  |  |
| 8 | >960 | >52 | >27 | >1.6 |  |  |  |
| 9 | >1080 | >58 | >31 | >1.8 |  |  |  |
| 10 | >1200 | ≥64 | >34 | >2.0 |  |  |  |
| 11 |  |  | >37 | >2.2 |  |  |  |
| 12 |  |  | >41 | >2.4 |  |  |  |
| 13 |  |  | >44 | >2.6 |  |  |  |
| 14 |  |  | >48 | >2.8 |  |  |  |
| 15 |  |  | >51 | >3 |  |  |  |
| 16 |  |  |  | >3.2 |  |  |  |
| 17 |  |  |  | >3.4 |  |  |  |
| 18 |  |  |  | >3.6 |  |  |  |
| 19 |  |  |  | >3.8 |  |  |  |
| 20 |  |  |  | >4 |  |  |  |

^1^Energy from SFA= Saturated fat content (g/100g)*9*4.18

^2^Oils derived from elements qualifying in the fruit and vegetable component qualify for fruit and vegetable component

A points= Points_energy_+ Points_Saturated fat_+ Points_Sugars_+ Points_Salt_

C points= Points_Fiber_+ Points_Protein_+ Points_F,V,P_

- If A points<7; Final nutritional score (FNS)=A points – C points
- If A≥7 FNS= A points – (Points_Fiber_ + Points_F,V,P_)

1. Beverages

| Component | A points | | | | | C points | | |
| --- | --- | --- | --- | --- | --- | --- | --- | --- |
| Points | Energy (kJ) | Saturated fat (g) | Sugars (g) | Salt (g) | NNS^1^ | Protein (g) | Fiber (g) | Fruits, vegetables, pulses (%) |
| 0 | ≤30 | ≤1 | ≤0.5 | ≤0.2 | Absence | ≤1.2 | ≤3 | ≤40% |
| 1 | >30 | >1 | >0.5 | >0.2 |  | >1.2 | >3 |  |
| 2 | >90 | >2 | >2 | >0.4 |  | >1.5 | >4.1 | >40% |
| 3 | >150 | >3 | >3.5 | >0.6 |  | >1.8 | >5.2 |  |
| 4 | >210 | >4 | >5 | >0.8 | Presence | >2.1 | >6.3 | >60% |
| 5 | >240 | >5 | >6 | >1 |  | >2.4 | >7.4 |  |
| 6 | >270 | >6 | >7 | >1.2 |  | >2.7 |  | >80% |
| 7 | >300 | >7 | >8 | >1.4 |  | >3 |  |  |
| 8 | >330 | >8 | >9 | >1.6 |  |  |  |  |
| 9 | >360 | >9 | >10 | >1.8 |  |  |  |  |
| 10 | >390 | >10 | >11 | >2.0 |  |  |  |  |
| 11 |  |  |  | >2.2 |  |  |  |  |
| 12 |  |  |  | >2.4 |  |  |  |  |
| 13 |  |  |  | >2.6 |  |  |  |  |
| 14 |  |  |  | >2.8 |  |  |  |  |
| 15 |  |  |  | >3 |  |  |  |  |
| 16 |  |  |  | >3.2 |  |  |  |  |
| 17 |  |  |  | >3.4 |  |  |  |  |
| 18 |  |  |  | >3.6 |  |  |  |  |
| 19 |  |  |  | >3.8 |  |  |  |  |
| 20 |  |  |  | >4 |  |  |  |  |

^1^NNS stands for Non-nutritive sweeteners, as listed in Appendix 2 of the EU Regulation n°1333/2008

A points= Points_energy_+ Points_Saturated fat_+ Points_Sugars_+ Points_Salt_+ Points_NNS_

C points= Points_Fiber_+ Points_Protein_+ Points_F,V,P_

- FNS=A points – C points

Nutri-Score allocation

| FNS | | | Class |
| --- | --- | --- | --- |
| Solid foods | Nuts, seeds, oils | Beverages |  |
| ≤0 | ≤-6 | Waters | A |
| 0 to 2 | -5 to 2 | ≤2 | B |
| 3 to 10 | 3 to 10 | 3 to 6 | C |
| 11 to 18 | 11 to 18 | 7 to 9 | D |
| ≥19 | ≥19 | ≥10 | E |
